# Wolff–Parkinson–White syndrome in a patient with type I Chiari malformation: management of an unusual association

**DOI:** 10.1097/MS9.0000000000005306

**Published:** 2026-07-15

**Authors:** Ghimire Sagun, Pahari Nabin, Pahari Mukesh, Mahat Muna, Maharjan Sunil, Maharjan Elina, Regmi Bibek

**Affiliations:** a Department of Neurosurgery, B&B International Hospital, Lalitpur, Nepal; b Department of Emergency Medicine and Critical Care, Mercy City Hospital, Butwal, Nepal; c Department of Medicine, Devdaha Medical College, Rupandehi, Nepal; d Department of Medicine, Heatherwood and Wexham Park Hospitals NHS Trust: Frimley Health NHS Foundation Trust, Ascot, UK; e Department of Medicine, Warrington and Halton NHS Trust, Warrington, UK; f Department of Surgery, University Hospital Southampton, Southampton, UK

**Keywords:** arrhythmias, case report, Chiari malformation type I, Nepal, syringomyelia, Wolff–Parkinson–White syndrome

## Abstract

**Introduction and importance:**

Wolff–Parkinson–White (WPW) syndrome is a congenital cardiac conduction disorder caused by an accessory pathway that predisposes patients to potentially life-threatening tachyarrhythmias. Chiari malformation type I is a congenital hindbrain anomaly characterized by the herniation of the cerebellar tonsils through the foramen magnum, often associated with syringomyelia and progressive neurological deficits. These two conditions are unrelated and rarely coexist; however, their concurrence presents significant anesthetic and perioperative challenges during neurosurgical procedures.

**Case presentation:**

A 21-year-old man presented with a 1-month history of lower back pain radiating to both lower limbs, accompanied by electric shock-like sensations, progressive weakness, and sensory loss. Magnetic resonance imaging revealed Chiari malformation type I with a large spinal syrinx, and posterior fossa decompression was planned. Routine preoperative electrocardiography unexpectedly demonstrated WPW syndrome. An electrophysiological study identified a right free-wall accessory pathway with inducible orthodromic atrioventricular re-entrant tachycardia. Successful radiofrequency catheter ablation eliminated preexcitation and rendered tachycardia non-inducible.

**Clinical discussion:**

WPW syndrome poses a substantial anesthetic risk due to the potential for perioperative life-threatening arrhythmias. Early detection and definitive treatment with catheter ablation enabled safe neurosurgical intervention. A multidisciplinary approach facilitated uncomplicated posterior fossa decompression, resulting in marked neurological improvement.

**Conclusion:**

This case underscores the importance of comprehensive preoperative assessment to identify rare but clinically significant comorbidities. Timely recognition and management of WPW syndrome allowed safe neurosurgical treatment, highlighting the critical role of multidisciplinary collaboration in optimizing patient outcomes.

## Introduction

Chiari malformation type I and Wolff–Parkinson–White (WPW) syndrome are two completely different clinical entities without any established clinical association. WPW is a cardiac conduction disorder with the possibility of life-threatening consequences such as supraventricular tachycardia and sudden cardiac death^[^1^]^. Whereas Chiari type I malformation is the elongation of the tonsils and the medial parts of the inferior lobes of the cerebellum into cone-shaped projections, which accompany the medulla oblongata into the spinal canal^[^2^]^. Along with this, in terms of clinical presentation as well, their respective signs and symptoms are completely different. Chiari malformation presents mostly as cough headache, oropharyngeal dysfunction, cranial nerve impairment, high intracranial pressure, peripheral motor deficit, and rarely acute impairment of cervical long tracts, with much rarer presentations such as quadriparesis or hemiparesis^[^3^]^. In contrast, in cases of WPW syndrome, asymptomatic presentation is more prevalent, and if symptomatic, then patients present with frequent paroxysmal episodes of tachycardia such as supraventricular tachycardia or atrial tachyarrhythmias, or both, and even the disastrous sudden cardiac death^[^4^]^. In both cases, surgical intervention is the treatment modality of choice for complete recovery^[^3,4^]^. There are only a handful of reported cases in the literature, where an incidental finding of Chiari malformation was observed in patients with WPW syndrome. Hence, despite the limited reporting of such an association, there might exist the possibility of a pathophysiological correlation, which needs to be delved into for optimal patient care. Here, we describe the presentation and management aspects in a case of concurrent association of two separate clinical conditions complicating each other’s management. This article has been reported in line with the TITAN 2025 checklist^[^5^]^. Our case report has been written in line with SCARE guidelines^[^6^]^.

HIGHLIGHTS

- An extremely unusual combination of Wolff–Parkinson–White (WPW) syndrome and type I Chiari malformation presented an unanticipated problem in a young adult.
- A routine preoperative electrocardiogramrevealed a previously unknown WPW pattern, altering the surgical approach completely.
- Targeted electrophysiological mapping and radiofrequency ablation eradicated the auxiliary circuit, allowing for safer neurosurgery.
- The posterior fossa decompression was carried out smoothly, resulting in a remarkable improvement in discomfort and lower-limb strength.
- This case demonstrates how rigorous examination and seamless collaboration across specialties may drastically improve patient outcomes.

## Case presentation

### Patient information

A 21-year-old man presented to the Neurosurgery Department of a tertiary care hospital in Nepal. The patient reported lower back pain of 1 month’s duration. The pain had an insidious onset, was dull in character, radiated to both lower limbs, worsened with physical activity, and was relieved by rest. It was associated with intermittent electric shock-like sensations in both lower limbs. He also complained of gradually progressive weakness in both lower limbs, accompanied by numbness. The patient reported a history of mild weakness in both lower limbs since the age of 6 years. There was no history of bowel or bladder incontinence. Antenatal, natal, and postnatal histories were unremarkable. He had no known medical or surgical illnesses. On further inquiry, the patient disclosed occasional episodes of palpitations occurring at rest. These episodes lasted approximately 30 seconds to 1 minute and resolved spontaneously. They were not associated with chest pain, shortness of breath, orthopnea, paroxysmal nocturnal dyspnea, or peripheral edema. During admission, all his vital signs were within normal limits. His cardiovascular examination was unremarkable, without any significant findings. Furthermore, his neurological examinations revealed muscle power of 4+ as per the Medical Research Council scale in bilateral lower limbs, with no other significant findings.

#### Investigations and management

**Figure 1.**
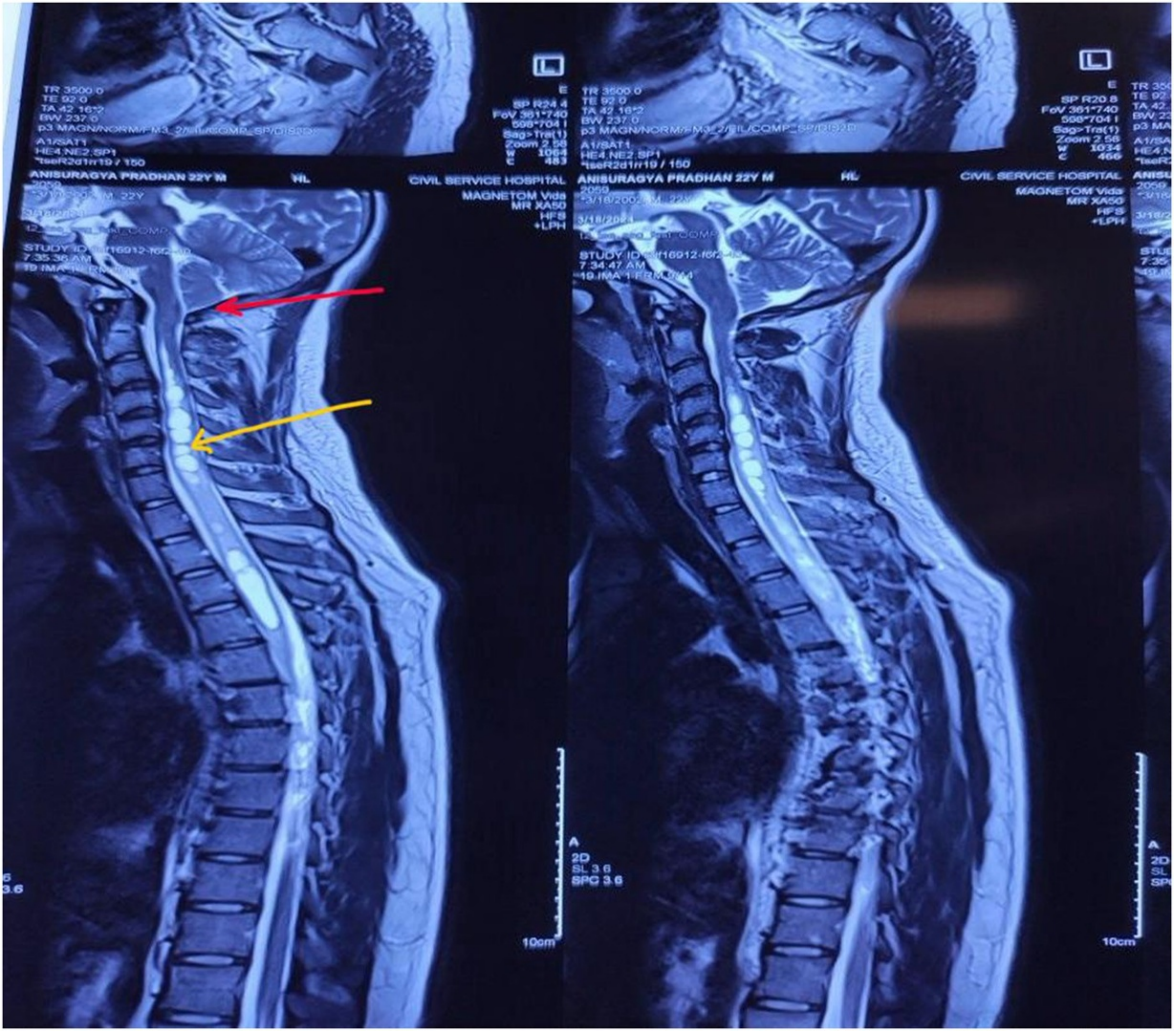
Non contrast MRI SPINE depicting comparison of syringomyelia pre operative vs post operative period with evident tonsillar herniation in pre operative image.

**Figure 2.**
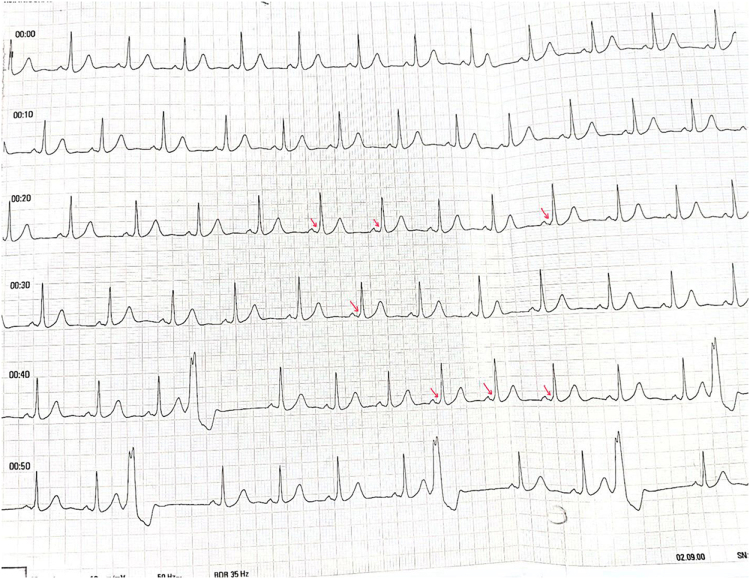
ECG suggestive of Wolff Parkinson white changes with evident delta wave.

Brain magnetic resonance imaging (MRI) showed signs indicative of type I Chiari malformation with significant syringomyelia (Fig. 1), aligning with his complaints of back pain and weakness in the lower limbs. He was scheduled for decompression of the posterior fossa. During the preoperative anesthetic assessment, his electrocardiogram (ECG) revealed indications of WPW syndrome (Fig. 2). Because of the cardiac risks linked to surgery, it was recommended that he undergo an electrophysiology study (EPS) with radiofrequency ablation (RFA) before the neurosurgical procedure.

EPS showed antegrade activation on baseline intracardiac recordings, suggesting a right-sided accessory pathway. Ventricular pacing exhibited eccentric activation of the coronary sinus with slight decrement, and the earliest atrial activation occurred at the proximal coronary sinus. Mapping of the tricuspid annulus identified the earliest atrial signal at the right free wall (9 o’clock position). Tachycardia could be induced, demonstrating retrograde activation at that identical location. A diagnosis of WPW syndrome with an accessory pathway on the right free wall and orthodromic atrioventricular reentrant tachycardia was established. RFA was conducted using a 7F sheath with an SL1 long sheath through the right femoral vein, along with extra diagnostic catheters positioned in the coronary sinus, right ventricle apex, and his bundle area. After the ablation, the delta wave vanished in seconds, ventricular pacing demonstrated ventriculoatrial dissociation, and tachycardia could no longer be induced.

#### Outcome and follow-up

**Figure 3.**
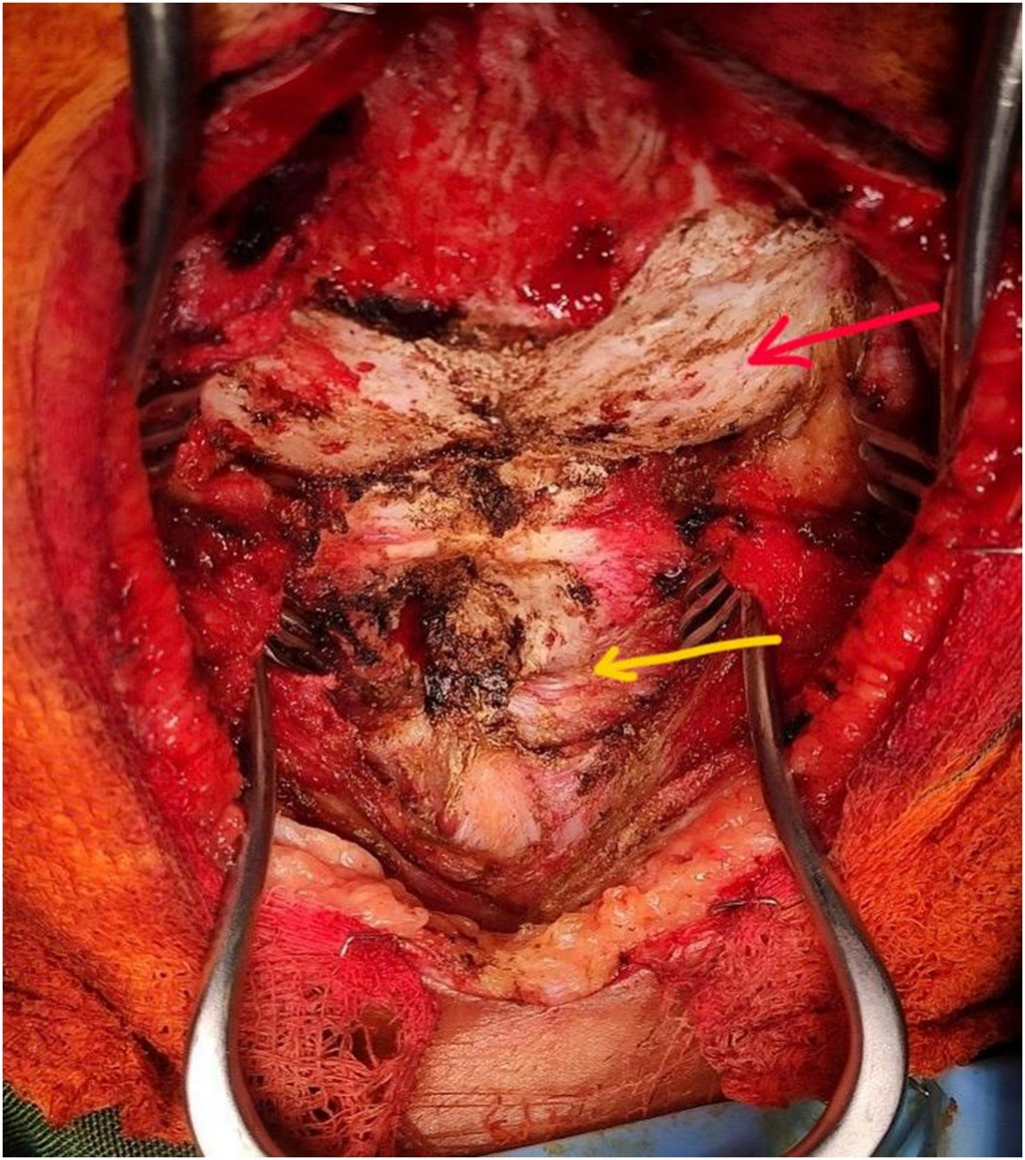
Intraoperative image depicting posterior fossa decompression surgery with exposed C1 and C2 arch.

The period following the ablation was unremarkable. The patient came back 2 weeks later for surgical treatment of his Chiari malformation. He later underwent posterior fossa decompression (Fig. 3). His recovery period after surgery was smooth, and he showed considerable progress in lower back pain and strength in both lower limbs. The patient was released 1 week post-surgery. The patient was on regular follow-up at our center, initially after 2 weeks, then after 3 and 6 months, during which no persistence of old complaints and no new deficits were noted. Along with this, his cardiac follow-up also showed resolution of WPW-like features in follow-up ECG, and there were no complaints of occasional tachypnea as well.

## Clinical discussion

Chiari 1 malformation, a common variant of Chiari malformations, encompasses a spectrum of hindbrain abnormalities affecting the cerebellum, brainstem, skull base, and cervical cord. The cerebellar tonsils of individuals with Chiari 1 malformation descend caudally through the foramen magnum, resulting in several neurological signs and symptoms^[^7^]^. Chiari malformations have been classified into four types, among which Chiari I is the least severe form that is frequently discovered incidentally. The cerebellar tonsils, not rounded, protrude 5 mm below the McRae Line^[^7^]^. The estimated prevalence rate for type I Arnold-Chiari malformation is 0.1–0.5%, less common in males, occurring in approximately 1 in every 1000 births^[^8^]^.

It is characterized by dizziness, syncopal attacks, progressive muscle weakness, stiffness of muscles, swallowing difficulties, vomiting, unsteady gait, difficulties in coordination, tinnitus, numbness, blurring of vision, and bowel and bladder dysfunction. It can, however, remain asymptomatic in some patients. These symptoms occur either due to the compression of the structures against the foramen magnum and spinal cord or due to syringomyelia^[^7^]^. MRI of the head and cervical spine is the investigation of choice for Chiari I malformation, demonstrating the cerebellar tonsillar descent. Myelography and X-ray of the neck and spine are other alternatives to MRI, which are not routinely used^[^7^]^. A widely accepted treatment involves surgically creating a decompression in the posterior cranial fossa through an occipital craniectomy. Although it enhances the disease’s underlying condition, it does not ensure symptom relief. These symptoms can be alleviated pharmacologically using non steroidal anti inflammatory drugs or muscle relaxants and exercise^[^9^]^.

WPW syndrome, a congenital condition, arises due to abnormal cardiac electrical conduction via an accessory pathway, resulting in arrhythmias that are potentially symptomatic and life-threatening. In sinus rhythm, the WPW pattern, or pre-excitation, is characterized by a short PR interval of less than 0.12 seconds, an initial slurred upstroke (delta wave) in the QRS complex, and a prolonged QRS duration of more than 0.12 seconds^[^10^]^. In this syndrome, impulses from the atria are abnormally transmitted to the ventricles via an accessory conducting pathway, which is situated between the atrial and ventricular walls and referred to as a bundle of Kent. A mutation in the PRKAG2 gene, which encodes a gamma-2 regulatory subunit of AMP-activated protein kinase, can lead to the inherited form^[^11^]^.

It is recommended to use the term WPW pattern in the absence of symptoms. Approximately one-third of the 0.15–0.25% of the population with the pattern will develop arrhythmias over a 10-year period. Between 0.8 and 1.9 life-threatening events per 1000 person-years occur, particularly in children^[^12^]^.

Patients with WPW syndrome have symptoms such as anxiety, palpitations, fatigue, dizziness, fainting, and shortness of breath. Approximately half of the patients who remain asymptomatic throughout their lives experience symptoms due to tachyarrhythmias, including atrial fibrillation, atrial flutter, paroxysmal supraventricular tachycardia, and, rarely, ventricular fibrillation and sudden death^[^13^]^.

In wide QRS complex arrhythmias, including atrial flutter, procainamide, sotalol, amiodarone, and flecainide are effective treatments. If the patients are unstable, immediate synchronized cardioversion should be performed, and catheter ablation of the accessory pathway is recommended. This definitive treatment for WPW disintegrates the abnormal electrical pathway while avoiding amiodarone, adenosine, beta-blockers, and calcium channel blockers, as these drugs isolate the accessory pathway and increase the risk of fatal arrhythmias like ventricular fibrillation by intensifying the ventricular rate. In patients with WPW syndrome who have atrial fibrillation or atrial flutter, atrioventricular (AV) nodal blocking drugs such as calcium channel blockers (verapamil and diltiazem), digoxin, adenosine, and beta-blockers should be avoided because they can increase conduction through the accessory pathway and precipitate ventricular fibrillation. If the patient is hemodynamically stable and does not exhibit pre-excited atrial fibrillation (normal QRS), AV nodal blockers may be used with caution; however, this is not generally suggested in classic WPW with pre-excitation. Useful drugs include procainamide, sotalol, amiodarone (IV, carefully monitored), and flecainide. In cases of multiple accessory pathways, Ebstein anomaly and hypertrophic cardiomyopathy should be evaluated^[^14–16^]^.

Chiari malformation and WPW syndrome are unrelated conditions. Furthermore, the literature review showed that there is only one reported case by Kruser *et al*, where the author describes a case of a 10-month-old child, a known case of WPW, who presented with syncope, and an incidental Chiari malformation was diagnosed. Later, the patient underwent posterior fossa decompression, but no symptomatic improvement was noticed; hence, a pacemaker was inserted, and finally, complete recovery was established. Correlating this with our case, we presume WPW must have been present as symptomatic since childhood, with incidental symptomatic Chiari malformation, unlike the case by Kruser *et al*^[^17^]^. Along with this, in such cases where significant cardiac comorbidity like WPW is diagnosed, major operative intervention should always be forfeited because there always exists the risk of the patient going into supra ventricular tachycardia, atrial fibrillation, and sudden cardiac death intraoperatively. Hence, to prevent such dreadful outcomes, risk stratification is mandated in such unusual associations^[^17^]^. For prompt and timely assistance in the diagnosis and management of such interrelated cases, the use of machine learning language models has been found to be useful as well^[^18^]^. Moreover, complete recovery is achievable only through surgical intervention in both instances. In this case report, we detail the approach for managing two interrelated yet distinct clinical conditions.

## Conclusion

This case underscores the rare combination of WPW syndrome and type I Chiari malformation. Timely detection of cardiac conduction issues during preoperative assessment is essential. The effective ablation of the accessory pathway facilitated a safe neurosurgical procedure, highlighting the significance of multidisciplinary collaboration in handling complex cases.

## Data Availability

Data sharing is not applicable to this article as no new data were created or analyzed in this study.
